# Effect of Excipients on the Quality of Drug Formulation and Immediate Release of Generic Metformin HCl Tablets

**DOI:** 10.3390/ph16040539

**Published:** 2023-04-04

**Authors:** Mosab Arafat, Molham Sakkal, Priya Yuvaraju, Anna Esmaeil, Vijo Poulose, Salahdein Aburuz

**Affiliations:** 1 College of Pharmacy, Al Ain University, Al Ain P.O. Box 64141, United Arab Emirates; mosab.arafat@aau.ac.ae (M.A.);; 2 Department of Pharmacology and Therapeutics, College of Medicine and Health Sciences, United Arab Emirates University, Al Ain P.O. Box 17666, United Arab Emirates; 3 Pharmalink and Medicina Group of Pharmacies, Abu Dhabi P.O. Box 41412, United Arab Emirates; 4 Department of Chemistry, United Arab Emirates University, Al Ain P.O. Box 17666, United Arab Emirates; 5 Department of Biopharmaceutics and Clinical Pharmacy, School of Pharmacy, The University of Jordan, Amman 11942, Jordan

**Keywords:** metformin, in vitro evaluation, thermal analysis, generic drug, brand-name drug, quality control test

## Abstract

Generic medications are bioequivalent to brand-name medications, but the quality and purity of generic medications are still debatable. The aim of this study was to compare the generic product of metformin (MET) to its branded counterpart using pure MET powder as a reference. Quality control tablet assessment and in vitro evaluation of drug release were carried out in various pH media. Additionally, several analytical methods and thermal techniques were used, namely differential scanning calorimetry (DSC), thermogravimetric analysis (TGA), X-ray diffraction (XRD), scanning electron microscopy (SEM), Fourier-transform infrared (FTIR), and confocal Raman microscopy. The results showed a significant difference between the two products. In terms of friability assessment, mean resistance force, and tablet disintegration, the generic MET product showed significant weight loss, higher mean resistance force, longer disintegration time, and a slower rate of drug release. In addition, DSC and TGA showed that the generic product had the lowest melting point and the least weight loss compared to the branded product and pure powder. XRD and SEM demonstrated some changes in the crystallinity structure of the molecule particles for the generic product. Additionally, FTIR and confocal Raman revealed the same peaks and band shifts in all samples, but with differences in the intensity for the generic tablet only. The observed differences could be due to the use of different excipients in the generic product. The possibility of forming a eutectic mixture between the polymeric excipient and metformin in the generic tablet was presumed, which might be attributed to alterations in the physicochemical properties of the drug molecule in the generic product. In conclusion, using different excipients might have a significant effect on the physicochemical properties of drugs in generic formulations, leading to significant changes in drug release behavior.

## 1. Introduction

In accordance with Food and Drug Administration (FDA) regulations and specifications [1], generic medication products are bioequivalent to their brand-name counterparts. However, variations in color, shape, excipients, impurities, residual solvents, and manufacturing procedures are allowed [1,2]. Certain generic drugs have been withdrawn from the market due to several issues, such as impurities, failure to meet dissolution testing requirements, and deviations from the required potency range [3,4,5]. Examples include metformin 500 mg tablets [3], quinapril 20 mg tablets, hydrochlorothiazide 12.5 mg tablets [5], anagrelide 1 mg capsules [6], paliperidone 3 mg extended-release tablets [7], tetracycline HCl 250 mg capsules [8], lidocaine HCl topical solutions, and thyroid tablets [9].

The immediate-release generic product of MET was chosen as the model generic drug in this study. The generic MET product was compared to the brand-name MET product using pure MET powder as a reference to identify any potential differences.

MET, also known as N, *N*-dimethylimidodicarbonicarbonidediamide, is a first-line medication for type 2 diabetes mellitus [10,11,12]. The molecular formula of MET is C_4_H_11_N_5_, and it has a molecular mass of 165.6 g/mol and a melting point range of 223–226 °C [12,13]. According to the biopharmaceutical classification system (BCS) classes of drugs, MET belongs to class III, indicating high solubility and low permeability [12,14,15]. Although MET is freely soluble in water (0.1 g/mL), it is only slightly soluble in alcohol and practically insoluble in acetone and methylene chloride [12,16]. MET has a LogP value of −0.5 and a pKa value of 12.4 [13,16].

Several research studies have suggested the possibility of excipient interference between the active ingredient and excipients in generic products [17,18,19,20]. Such interference could affect the physicochemical properties of the active ingredient, including disintegration time, drug release rate, and shelf life [17,18,19,20]. Diluent excipients, such as microcrystalline cellulose and lactose, have been reported to be incompatible with L-phenylalanine amine [21] and ketoprofen [22]. Similarly, some lubricants, such as magnesium stearate and talc, can cause degradation or discoloration of the formulation when mixed with certain active ingredients, such as ibuprofen, acetylsalicylic acid, and quinapril [23,24]. Additionally, binders, such as starch, might cause degradation of hydralazine HCl [25]. Moreover, polyethylene glycol has been shown to form a eutectic mixture with ibuprofen, altering drug release profile [18]. Similarly, the formation of a eutectic mixture of felodipine and polyvinylpyrrolidone has been shown to impact the drug stability of the final product [19]. Finally, the combination of hydroxypropyl methylcellulose and poloxamer-188 has been shown to delay the drug release of diltiazem hydrochloride by forming a matrix system [26].

A number of analytical techniques have been found to be essential in the evaluation of potential variations between generic and branded medications [27,28,29], such as: thermal gravimetric analysis (TGA), differential scanning calorimetry (DSC) [30,31,32], Fourier-transform infrared (FTIR), confocal Raman microscopy [33,34], X-ray diffraction (XRD), and scanning electron microscopy (SEM) [34,35]. For instance, one study reported incompatibility between the active ingredient ibuprofen and the excipients polyvinylpolypyrrolidone and polyvinylpyrrolidone K30 using thermal techniques [28,29,36]. In other research studies, XRD and DSC/TGA have been used to identify low amounts of the active ingredients in some generic drugs containing acetaminophen [36,37]. Another study showed changes in the properties of sildenafil caused by excipients, such as butylated hydroxyanisole, ascorbic acid, and citric acid, using DSC and FTIR [28].

The aim of this work was to investigate possible differences between the branded drug product of MET tablets (850 mg) and the generic products of MET tablets (850 mg) using pure MET crystal powder as a reference for drug identification. Accordingly, a series of experiments was conducted to determine the influence of different incorporated excipients on the physicochemical properties of generic MET tablets. To achieve these objectives, a variety of analytical methods and thermal techniques were used, namely DSC, TGA, XRD, SEM, FTIR, and confocal Raman microscopy. In addition, the in vitro drug release rate was evaluated, and quality control tests for the tablets were determined. This study was the first of its kind to investigate the potential causes of issues associated with generic MET products. In this study, a number of thermal and analytical techniques were used as scanning tools prior to bioequivalence assessment [38,39].

## 2. Results

### 2.1. Quality Controlled Tablet Assessment

Quality control tests showed similarities between the generic MET product and the branded MET product tablets (850 mg), except for friability, mean resistance force, and disintegration time for the two products, as shown in Table 1. Both products met the USP friability criteria of less than 1% weight loss, but the generic MET product showed 0.04% more weight loss (*p* < 0.05). The mean resistance force was significantly higher for the generic MET product (450.90 N ± 53.24 N) compared to the branded MET product (299.10 N ± 44.71 N) (*p* < 0.05). The generic MET product took 5 min longer to completely disintegrate compared to the branded MET product, which is significantly higher (*p* < 0.05).

### 2.2. In Vitro Dissolution Drug Release Evaluation

**Figure 1:**
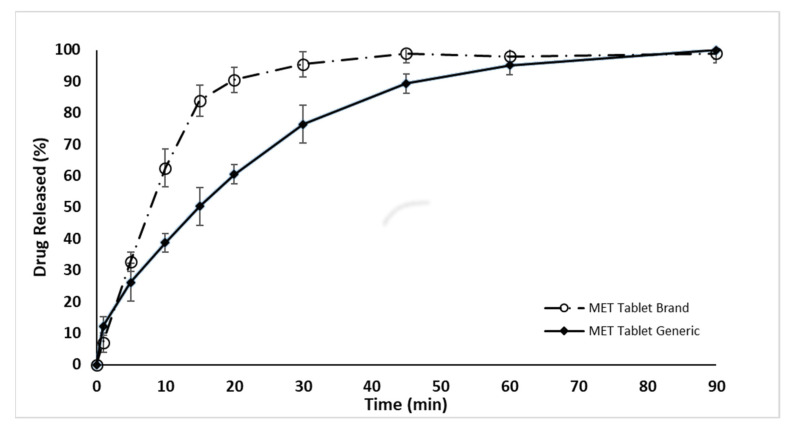
The dissolution release rate for the generic MET product (850 mg) compared to the branded tablet over 90 min in distilled water at 37 °C. Values are means ± S.D. (*n* = 6).

**Figure 2:**
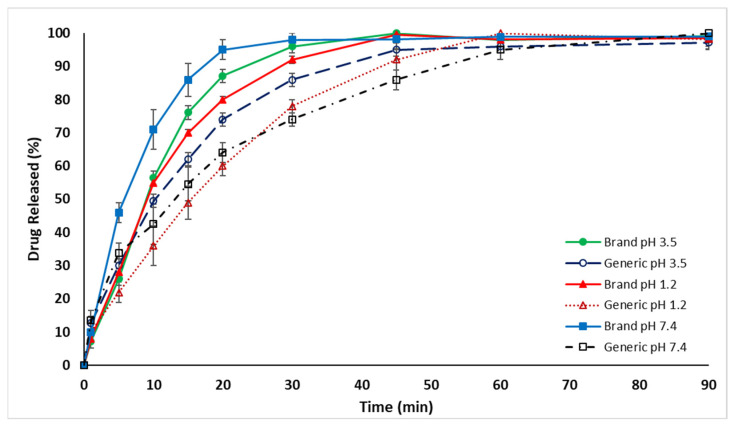
The dissolution release rate of generic MET tablets (850 mg) compared to branded MET tablets over 90 min, using different pH incubation media (pH 1.2, 3.5, and 7.4) at 37 °C. Values are means ± S.D. (*n* = 6).

**Figure 3:**
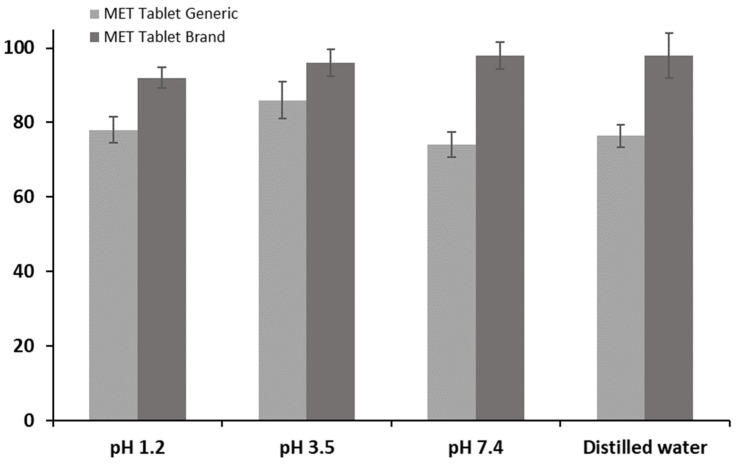
The drug release percentage of generic MET tablets (850 mg) compared to branded MET tablets at 30 min incubated in different pH media (pH 1.2, 3.5, 7.4) at 37 °C. Values are means ± S.D. (*n* = 6).

The drug release rate for the generic MET product (MET Tablet Generic) was found to be lower than that of the branded MET product in distilled water (DW), as illustrated in Figure 1. The results showed that the percentage of drug release from the generic MET product was below 80% in the first 30 min, which did not meet the standards and specifications set by the United States Pharmacopeia (USP) [40,41,42], whereas 95% of the branded MET product (MET Tablet Brand) was released in DW in the first 30 min. Figure 2 and Figure 3 demonstrate that the drug release rate of the generic MET product was significantly lower in all pH conditions (1.2, 3.5, and 7.4) and particularly over the first 30 min compared to the branded MET product (*p* < 0.05). It is evident that the release rate of the generic MET product was dependent on the pH media, as compared to the branded MET product, which was pH-independent [43].

### 2.3. Differential Scanning Calorimetry

**Figure 4:**
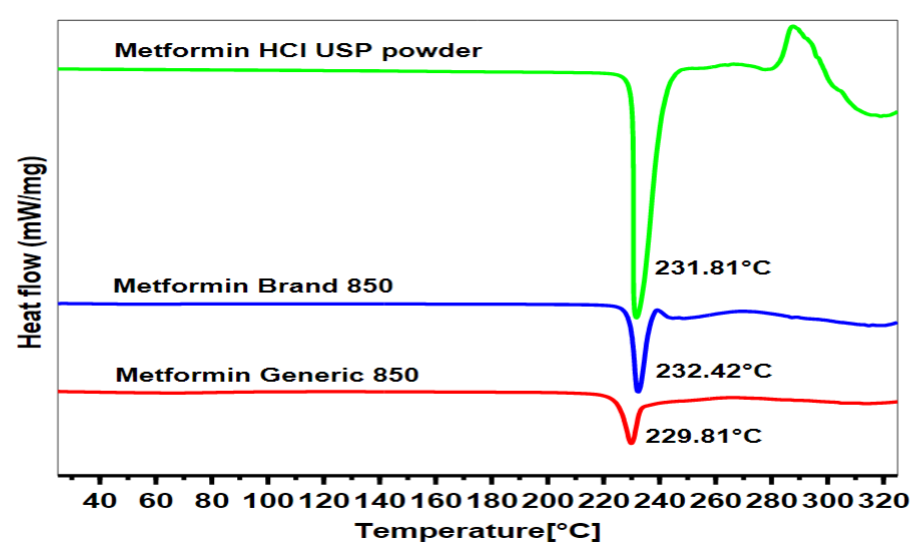
DSC thermal analytical spectra of MET in the form of drug pure powder and branded and generic tablets.

Figure 4 presents the results of the thermal profiles for the pure MET powder, the MET branded product, and the MET generic product, as determined by DSC. The thermal profile of the branded MET product displayed an endothermic peak at 232.42 °C, with a range from 224.22 °C to 239.42 °C, which is very similar to the endothermic peak value for the pure MET powder. The endothermic peak of pure MET powder was 231.81 °C, with a range from 217.03 °C to 248.79 °C. On the other hand, the generic MET product had the shortest endothermic peak at 229.81 °C, with the sample starting to melt at 204.89 °C and ending at 246.86 °C. Furthermore, the area under the curve was the lowest for the generic MET product; the heat absorbed by the samples was 165.65 J/g for the generic MET product, 226.21 J/g for the branded MET product, and 324.77 J/g for pure MET powder (*p* < 0.05).

### 2.4. Thermal Gravimetric Analysis

**Figure 5:**
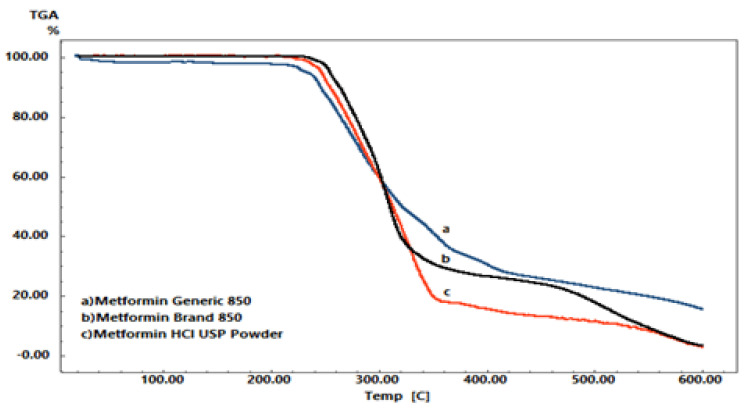
TGA thermal analytical spectra of MET in the form of (**a**) generic tablet, (**b**) branded tablet and (**c**) drug pure powder.

The TGA profiles for the three samples (pure MET powder, branded MET product, and generic MET product) showed significant differences in terms of weight loss (*p* < 0.05). As shown in Figure 5, the weight losses for pure MET powder and the branded MET product were almost the same, with approximately 97% of the total weight loss. The weight loss was 7.937 mg for the pure MET powder and 6.114 mg for the branded MET product. However, the generic MET product showed a significantly lower amount of weight loss, with approximately 82% of the total weight lost (*p* < 0.05), equaling 4.936 mg. The onset temperature of weight loss also varied among the samples as shown in Figure 5. These variations in weight loss and (*T*_onset_) temperature indicate that some differences occurred in the physical properties of the generic MET product.

### 2.5. X-ray Diffraction

**Figure 6:**
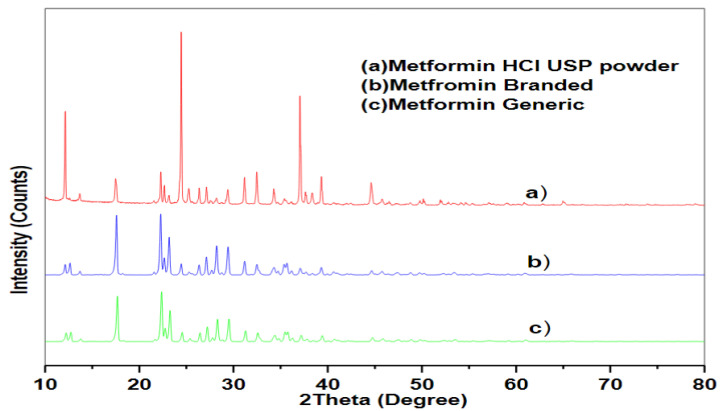
X-ray diffraction analytical spectra of MET in the form of (**a**) drug pure powder, (**b**) branded tablet and (**c**) generic tablet.

Figure 6 shows the diffraction peak patterns for pure MET powder (a), the branded MET product (b), and the generic MET product (c), which describes the clear atomic arrangements and physical states of the samples. The diffraction peaks were located at nearly the same positions for the three samples. However, the generic MET product exhibited a lower intensity of peaks at 2θ angles of 17°, 22°, 23°, and 31° compared to the branded MET product. This difference in peak intensity might suggest a possible variation in the crystalline structures of the two products.

### 2.6. Scanning Electron Microscopy

**Figure 7:**
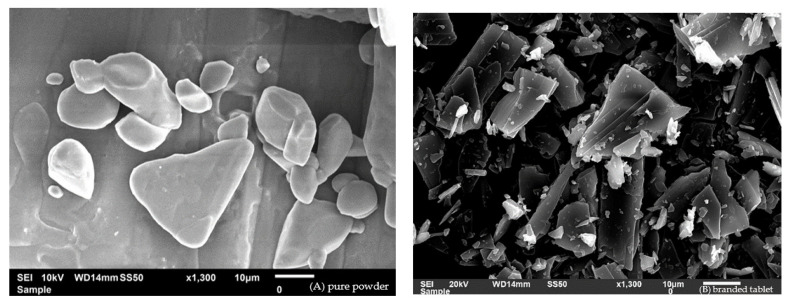

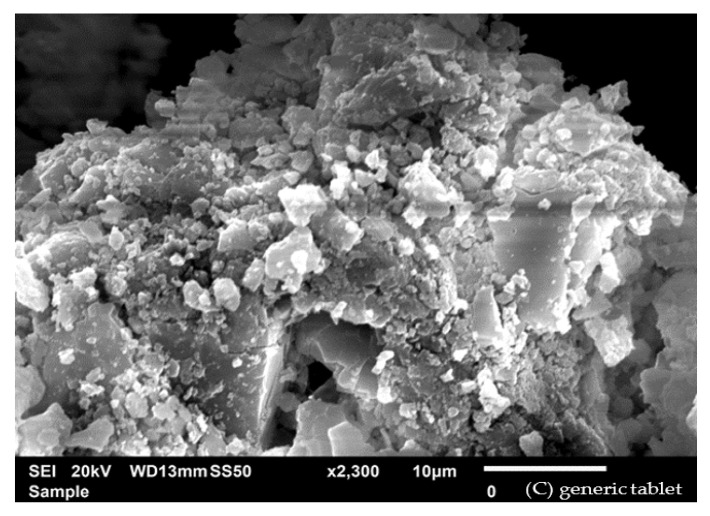
SEM images of MET in the form of (**A**) drug pure powder, (**B**) branded tablet, and (**C**) generic tablet.

The main morphological features of pure MET powder, the branded MET product, and the generic MET product are presented in Figure 7. The particle size was the smallest for the generic MET product, followed by the branded MET product and then pure MET powder. The distance between particles in the generic MET product was the shortest in comparison to the two other samples. The SEM images of the three samples showed a coarse surface for the particles of the generic MET product, with some cracks and stretches, while a smooth surface was observed for the branded MET product and pure MET powder.

### 2.7. Fourier-Transform Infrared Spectroscopy

**Figure 8:**
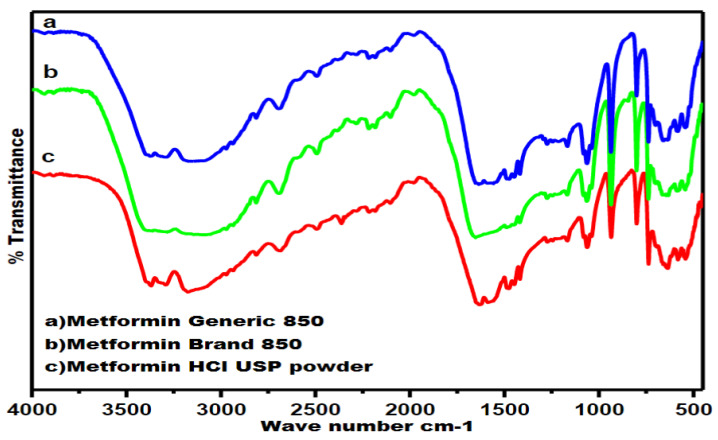
Fourier-transform infrared analytical spectra of MET in the form of (**a**) generic tablet, (**b**) branded tablet and (**c**) drug pure powder.

**Figure 9:**
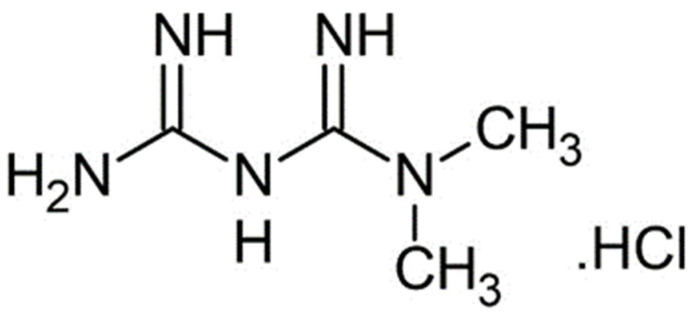
Chemical structure of Metformin HCl.

Figure 8 shows the FTIR absorption bands for pure MET powder, the branded MET product, and the generic MET product. The chemical structure of the active ingredient MET is presented in Figure 9. By comparing the spectra of the three samples, the presence of certain bands without shifting may indicate compatibility between excipients and drug powder in the tablet formulations. All three samples showed characteristic bands of the MET compound in their spectra, but the intensity of the peaks varied between the three samples. The fingerprint region below 1500 cm^−1^ had similar bands at the same wave number with slight variation in peak intensity. For example, the -N-H waging was reflected by a sharp peak at 764 cm^−1^ in the spectrum.

The diagnostic region of the FTIR spectrum, which is particularly useful in identifying functional groups, revealed several peaks in all three samples, with different intensities for the three samples. The peaks at 1571 cm^−1^, 1625 cm^−1^, and 3170 cm^−1^ correspond to the stretching vibrations of C-N, C=N, and N-H, respectively. Furthermore, the 3298 cm^−1^ band is attributed to a secondary amine, while the 3369 cm^−1^ band indicates a primary amine functional group.

### 2.8. Raman Spectroscopy

**Figure 10:**
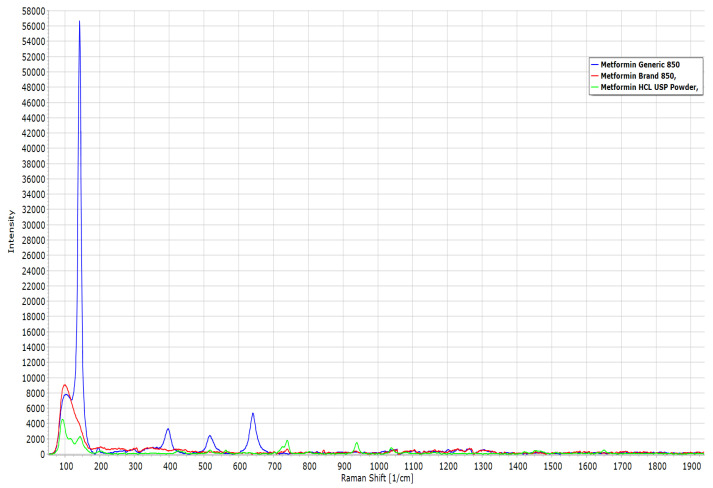
Raman spectroscopy of MET in the form of pure powder, branded tablet, and generic tablet.

Figure 10 presents the results of confocal Raman analysis for pure MET powder, the branded MET product, and the generic MET product. Most of the peaks in the Raman spectra appeared in the same positions for the three products, but the intensity of the peaks varied between the different products, with the highest intensity observed for the generic MET product. Table 2 presents the main peaks observed in the Raman spectra, along with the responsible functional group and the intensity of each peak.

## 3. Discussion

The study was able to identify variations between the generic and branded MET products by utilizing a number of thermal and analytical techniques. The methods utilized in this study can be applied to other generic medications to evaluate their equivalence to branded products. This study highlights the potential of using thermal and analytical techniques to provide valuable information about the quality of medications prior to the required bioequivalence assessments.

The higher values observed for the mean resistance force, tablet disintegration, and friability in the generic MET product compared to the branded MET product might be due to the incorporation of starch in the tablet formulation of the generic MET product, as reported by some research studies [44,45]. This ingredient has been known to impact the properties of tablets and may play a role in the differences observed between the two products [40,46,47]. Starch is a hygroscopic material that may increase a tablet’s absorption of moisture, leading to a more compact tablet with a higher mean resistance force [46,47]. Additionally, the hydrophilic nature of starch can affect the disintegration period of a tablet [44,45]. Starch molecules absorb moisture and form a gel-like substance that binds particles together, which might prolong the release rate of active ingredients [44,45]. Similar effect of starch on the hydralazine HCl was reported [25].

The slower release rate of the generic MET product compared to the branded MET product in all dissolution media (DW, pH 1.2, pH 3.5, and pH 7.4) is likely due to differences in the incorporated excipients in the formulation, as reported in various research studies [44,45,48,49,50,51]. The generic MET product contains microcrystalline cellulose, polyethylene glycol (PEG), starch, talc, and titanium oxide [44,45,50]. Microcrystalline cellulose is a highly compressible and porous excipient that can create a physical barrier around the active ingredient, thereby slowing down the release rate of the drug molecule [48,49,52]. Polyethylene glycol and starch are hydrophilic excipients that can absorb water and form gel-like substances, leading to a significant delay in drug release if present in a high percentage [44,45]. Additionally, talc and titanium oxide are hydrophobic excipients with large particle sizes that can physically obstruct the release of active ingredients, leading to a slower release rate [41,44]. The results of our study are consistent with previous literature that demonstrated that PEG can form a eutectic mixture with ibuprofen, resulting in slower drug release [18]. Additionally, another study reported that PEG can cause changes in the disintegration profile of glimepiride from the formulation [48].

The lower heat absorbed and lower amount of weight lost by the generic MET product compared to the branded MET product, as shown in the DSC and TGA thermal profiles, could be due to several possibilities, as has been reported in other research studies [53,54,55]. One of the possibilities might be the presence of different excipients in the generic MET product, such as polyethylene glycol (PEG) [53], which is commonly used as a binder or stabilizer and may result in lower heat absorption during DSC analysis and a reduced amount of weight loss during TGA analysis [53]. Talc and titanium oxide may also have significant impacts on the crystalline form of the active ingredient and may lead to alterations in the physical properties of the formulation and the thermal profile of the drug molecule [54]. Our observations are supported by previous studies that have shown that PEG can maintain temperature during drug release timing [56].

Another possibility could be due to the formation of a eutectic mixture between hydrophilic polymeric components, such as PEG or starch, and the active ingredient MET [55], which may have an impact on the drug’s thermal profiles [55]. Some eutectic mixtures may lead to separate transitions for each component instead of a single solid-to-liquid transition, resulting in a lower melting point [57,58]. Additionally, during TGA analysis, the possibility of changing in the generic MET product might cause a lower melting point, and the thermal degradation of the material would be less severe, resulting in a lower overall weight loss compared to pure MET powder or the branded MET product [55,57]. This was supported by a slowdown in the drug release rate of the generic MET product compared to the branded MET product. This slowdown in the drug release rate is a result of increased viscosity due to the formation of a eutectic mixture [55,58].

The observed decrease in the intensity of the XRD peaks for the generic MET product compared to the branded MET product might indicate changes in the crystal structure of the generic MET product. This alteration might be related to differences in the incorporated excipients in the two products, as suggested by previous research studies [59,60]. A previous study reported that microcrystalline cellulose can function as a crystal growth inhibitor, resulting in the formation of smaller and less ordered crystals that scatter X-rays less effectively. This may account for the observed decrease in the XRD peaks [59]. This is supported by the SEM results, in which the crystal structure of the generic MET product was smaller and less organized than the branded MET product [60]. Additionally, these smaller crystals may then pack together more tightly, which would explain the short distance between particles in the generic MET product [60].

Several main bands in FTIR and confocal Raman were similar for all samples but with different peak intensities. For instance, the C-N, C=N, and CH functional groups were indicated by the presence of wave numbers 1571 cm^−1^, 1625 cm^−1^, and 3170 cm^−1^ in FTIR, respectively, whereas they appeared at shift numbers 1040 cm^−1^, 1650 cm^−1^, and 2850 cm^−1^ in confocal Raman for all three products. The existence of these functional groups confirms the presence of the MET compound in the formulation of both products, yet the intensity of the peaks varied between the branded MET product and the generic MET product. The variations in peak intensities observed in the FTIR and Raman spectra of the three products can be attributed to differences in the amount of active ingredient dispersed in the three samples [61,62,63]. The different tablet sizes of the generic and branded MET products resulted in different amounts of the MET drug molecule present in the samples used for FTIR and confocal Raman analysis. However, the peak intensity was found to be highest for pure MET powder since the sample only contained the MET drug molecule. This observation is in line with previously reported studies [61,62,63].

## 4. Material and Methods

### 4.1. Materials

The brand-name MET tablets (850 mg) and the generic MET tablets (850 mg) were purchased from Boots Pharmacy (Dubai, United Arab Emirates). The pure MET powder was obtained from Sigma-Aldrich (St. Louis, MO, USA).

### 4.2. Weight Variation of the Tablets

A sensitive digital scale (Shimadzu, Kyoto, Japan) was used to weigh a total of 20 tablets for each product. The tablets were individually weighed to determine their average weight. The weighing procedure followed the guidelines and standards set by the United States Pharmacopeia (USP) [64], ensuring that the measurements were performed in accordance with established regulatory requirement.

### 4.3. Tablet Friability Assessment

The friability test was conducted in accordance with the standards and specifications set by the USP [38]. For each product, 10 tablets were selected, dusted, and weighed before being placed into the drum of a friabilator TA 220 (Erweka GmbH, Heusenstamm, Germany). The tablets were then subjected to the friabilator’s revolving drum for 4 min at a speed of 25 RPM, after which they were removed and visually inspected for cracks or broken edges. Any loose particles were carefully removed by dusting before the tablets were weighed once more to calculate the percentage of weight loss.

### 4.4. Mean Resistance Force of the Tablets

The mean resistance force of the two products was determined using a TBH-225 TD hardness tester (Erweka GmbH, Heusenstamm, Germany). Ten tablets each of the branded MET product and the generic MET product were randomly selected for the hardness assessment. The standard deviation was calculated for each product, and the mean resistance force of the tablets was measured. The mean resistance force test was performed in accordance with the guidelines and specifications set by the USP [64].

### 4.5. Chemical Content of the Drug

The chemical composition of the two products was determined by weighing and triturating 20 tablets from each product. A solution of MET was then prepared by diluting 100 mg of the sample in 100 mL of distilled water (DW). The prepared solution was further diluted and filtered to achieve a final concentration of 100 µg/mL. The concentration of the solution was measured using spectrophotometry at an ultraviolet (UV) wavelength of 233 nm, and the entire process was conducted six times (*n* = 6) for each product to ensure the accuracy and precision of the assessment.

### 4.6. Tablet Disintegration

The disintegration timing of the tablets for both products was measured using a fully automated disintegration instrument (PTZ Auto EZ, Hainburg, Germany). The disintegration test was performed using DW at a fixed temperature of 37 °C ± 0.5 °C for a duration of 30 min. Six tablets were randomly selected from each product and subjected to the disintegration test. The disintegration time of all the tablets was accurately recorded for analysis. The assessment was performed in accordance with the specifications set by the USP.

### 4.7. In Vitro Dissolution Evaluation

In vitro drug release evaluations for both products were performed using a Dissolution Apparatus II model Dis 8000 (Copley Scientific, Nottingham, UK). The apparatus was operated at a speed of 100 RPM, and the temperature of the dissolution medium was maintained at 37 °C ± 0.5 °C. The in vitro dissolution evaluation was carried out using 900 mL of filtered DW, as well as three different pH media values (pH 1.2, pH 3.5, and pH 7.4), to simulate various physiological pH conditions. Samples of 5 mL were collected from each vessel at specific time intervals (0, 5, 10, 15, 20, 30, 45, 60, and 90 min). The samples were diluted using the same incubation media, followed by filtration of the samples. The filtered samples were then analyzed using UV spectrophotometry at a wavelength of 233 nm. The experiment was conducted for six samples of each product (*n* = 6). The in vitro dissolution evaluation was conducted in accordance with the specifications set by the USP.

### 4.8. Differential Scanning Calorimetry

The DSC measurements of the two products and pure MET powder were performed using a DSC-60 Plus instrument (Shimadzu, Kyoto, Japan). For each product, a precise amount of 3–5 mg powder was weighed and loaded into sample pans for thermal analysis. The scanning temperature for each sample was performed between 25 and 350 °C at a rate of 10 °C per min, while a constant flow of nitrogen at 100 mL/min was maintained in a controlled environment. LabSolutions TA software was used to process and analyze the obtained results. The experiment was performed on six samples of each product (*n* = 6) to ensure the statistical validity of the results.

### 4.9. Thermogravimetric Analysis

TGA of the two products and pure MET powder was performed using a TGA-50 instrument (Shimadzu, Kyoto, Japan). For each sample, 10–15 mg of powder was accurately weighed and placed in an alumina pan. The samples were scanned over a temperature range of 0 °C to 600 °C at a rate of 15 °C per min while being exposed to a nitrogen flow rate of 50 mL per min. The analysis process was closely monitored and controlled using LabSolutions TA Thermal Analysis Workstation software. To ensure the statistical validity of the results, the experiment was conducted using six samples of each product (*n* = 6).

### 4.10. X-ray Diffraction

The crystalline structure of both products and pure MET powder was determined using an XRD 6100 (Shimadzu, Kyoto, Japan). The XRD patterns of the samples were obtained by conducting 2θ scanning within a range of 10° to 80° at a rate of 2° per min. The analysis process was carefully maintained and conducted consistently to ensure high-quality results [65].

### 4.11. Scanning Electron Microscopy

The morphological features of the two products and pure MET powder were examined using a JSM-6010PLUS/LA Scanning Electron Microscope (JEOL, Tokyo, Japan). To prepare the samples for testing, a small amount was attached to the specimen holder stub using double-coated adhesive carbon tape. Before conducting the test at 20 kilovolts, a layer of gold coating was applied to the sample for 10 min using a Cressington 108 Auto Sputter Coater. The gold-coated sample was then placed in the sample stage and analyzed using InTouchScope SEM software. The process was carried out with precision to ensure the quality of the analysis.

### 4.12. Fourier-Transform Infrared Spectroscopy

To obtain the FTIR spectrum profiles of the two products and pure MET powder, a Thermo Nicolet Nexus 670 spectrometer (GMI, Ramsey, NJ, USA) was utilized. The samples were first mixed with dry potassium bromide at a weight ratio of 1:100 to create pellets. The transmittance of the samples was then recorded within the range of 4000 cm^−1^–450 cm^−1^. In order to obtain the spectra, 32 scans were conducted, and the data were processed using OMNIC 9 software to ensure the accuracy and consistency of the obtained results [66,67].

### 4.13. Confocal Raman Microscopy

A Confocal Microscope Raman/PL System (NOST, Daejeon, Republic of Korea) was utilized to obtain the Raman spectra for the two products and pure MET powder. To conduct Raman mapping, the sample was mounted on a glass slide and subjected to a five-sec laser exposure using a 20× objective lens. The Raman shift range of each sample, spanning from 0 cm^−1^ to 4000 cm^−1^, was scanned, and the corresponding counts were recorded. The RAON-SPEC program was used to analyze all samples.

### 4.14. Statistical Analysis

The statistical analysis of variance was performed using the independent sample *t*-test to compare the mean values of the determined variables. Differences were considered significant if *p* < 0.05 [68,69]. The software utilized for the analysis was the Statistical Package for the Social Sciences (SPSS) Version 26.

## 5. Conclusions

In conclusion, some variations between the generic MET product and the branded MET product were observed with respect to friability, mean resistance force, disintegration timing, in vitro drug release rate, thermal profile, morphological features, and peak intensities. These variations might be attributed to excipient differences in the formulation of the generic tablet. A possible interferences between excipients and active ingredients was presumed. Effect of excipients on active ingredients of MET might have an effect on the drug, resulting in a decrease in the drug release rate for the generic MET tablets.

## Figures and Tables

**Table 1:** Quality control test results for generic MET tablets compared to branded MET tablets.

Measurements	Generic Tablet	Branded Tablet
Average Weight (mg)	850 ± 0.2	850 ± 0.1
Weight Variation Range (%)	0.98 ± 0.02	0.91 ± 0.01
Tablet Friability (%)	0.16 ± 0.02 *	0.12 ± 0.01
Mean Resistance Force (N)	450.90 ± 53.24 *	299.10 ± 44.71
Mean Tablet Length (mm)	8.98 ± 0.32 *	13.56 ± 0.16
Chemical contents (%)	1.39 ± 0.20	1.53 ± 0.13
Disintegration time (min)	15.25 ± 0.57 *	10.30 ± 0.14

Significant difference marked with * when (*p* ≤ 0.05) compared to branded MET tablet.

**Table 2:** Peak intensities of pure MET powder and branded and generic tablets.

Raman-Shift (cm^−1^)	MET Generic	MET Brand	MET Powder	Functional Group
65	8000	9000	4500	C-N-C deformation
130	57,000	4100	2200	C-N-C deformation
400	3000	1000	500	C-N-C deformation
880	500	500	1900	N-H wagging
1040	1000	1000	1000	C-N stretching
1650	100	100	100	C=N stretching
2850	200	220	240	CH_3_ sym. stretching
2950	400	400	500	CH_3_ asym. stretching

## Data Availability

Data is contained within the article.
